# Evaluating novel synthetic compounds active against *Bacillus subtilis* and *Bacillus cereus* spores using Live imaging with SporeTrackerX

**DOI:** 10.1038/s41598-018-27529-4

**Published:** 2018-06-14

**Authors:** Soraya Omardien, Alexander Ter Beek, Norbert Vischer, Roy Montijn, Frank Schuren, Stanley Brul

**Affiliations:** 1 Swammerdam Institute for Life Sciences, Department of Molecular Biology and Microbial Food Safety, University of Amsterdam, Science Park 904, 1098 XH Amsterdam, The Netherlands; 2 Swammerdam Institute for Life Sciences, Department of Bacterial Cell Biology and Physiology, University of Amsterdam, Science Park 904, 1098 XH Amsterdam, The Netherlands; 3 Microbiology and Systems Biology Group, TNO, Utrechtseweg 48, 3704HE Zeist, The Netherlands

## Abstract

An empirical approach was taken to screen a novel synthetic compound library designed to be active against Gram-positive bacteria. We obtained five compounds that were active against spores from the model organism *Bacillus subtilis* and the food-borne pathogen *Bacillus cereus* during our population based experiments. Using single cell live imaging we were able to observe effects of the compounds on spore germination and outgrowth. Difference in sensitivity to the compounds could be observed between *B*. *subtilis* and *B*. *cereus* using live imaging, with minor difference in the minimal inhibitory and bactericidal concentrations of the compounds against the spores. The compounds all delayed the bursting time of germinated spores and affected the generation time of vegetative cells at sub-inhibitory concentrations. At inhibitory concentrations spore outgrowth was prevented. One compound showed an unexpected potential for preventing spore germination at inhibitory concentrations, which merits further investigation. Our study shows the valuable role single cell live imaging can play in the final selection process of antimicrobial compounds.

## Introduction

To survive harsh or nutrient-free environmental conditions, spore-forming bacteria undergo sporulation that results in the formation of dormant spores that are metabolically inactive and partially dehydrated due to the replacement of water by Ca^2+^-dipicolinic acid. Dormant spores are resilient and can withstand conditions such as high temperatures, radiation, toxic chemicals or desiccation^1,2^. They become vulnerable when germination occurs, which is generally triggered by favourable environmental conditions. Gram-positive non-pathogenic *Bacillus subtilis* is often used as a model organism to understand the sporulation process of pathogenic spore-formers such as those within the *Bacillus cereus* family (*B*. *cereus*, *Bacillus anthracis*, *Bacillus thuringiensis*, *Bacillus mycoides*, *Bacillus pseudomycoides and Bacillus weihenstephanensis*)^3^. While genotypically distant, the only major phenotypical difference between *B*. *subtilis* dormant spores and the *B*. *cereus* family is the encasement of the spore-coat, which consists of an inner coat, outer coat and crust. The spore-coat of *B*. *cereus*, and related members, is surrounded by an additional layer, known as the exosporium, which mediates the binding of the bacterium to surfaces^4^.

*B*. *cereus* strains are responsible for two types of food-borne illnesses: the emetic and diarrheal syndrome^4^. In addition to its link to food-borne illnesses, *B*. *cereus* is also considered a medically relevant pathogen^5^. Molecularly it is difficult to distinguish between *B*. *cereus*, *B*. *anthracis* or *B*. *thuringiensis*^3^. *B*. *anthracis* can only be distinguished by the presence of plasmids, pXO1 and pXO2, involved in the production and regulation of the anthrax virulence factors, tripartite toxin and the capsule, respectively^3,6,7^. *B*. *thuringiensis* can only be distinguished by the formation of protein crystal inclusions, protoxins, during sporulation^3^. Therefore, *B*. *cereus* can be considered a model organism to understand the behaviour of *B*. *anthracis* and *B*. *thuringiensis*.

In our study, we aimed to address the rise in resistance development by searching for novel antimicrobials active against Gram-positive spore-forming bacteria that might have subsequent application in the food or clinical sector. A combinatorial chemistry approach was employed to obtain from Pyxis Discovery B.V. (Delft, the Netherlands) a synthetic compound library active against Gram-positive bacteria. Their selection approach utilizes computational software to screen compounds for pre-determined characteristics, such as the lack of metals or reactive groups, novelty, and to what degree the compounds under study are heterocyclic^8^. Heterocyclic compounds are well known for their therapeutic potential as antimicrobial agents against various microorganisms. For instance thiazoles^9^, oxadiazoles^10^, triazoles^11^, triazolothiadiazines^12^ and benzophenones^13^ have shown antibacterial, antifungal or antiviral properties. The original library of around 2000 compounds was screened for activity against Gram-positive bacteria in a population based assay against the model organism *Staphylococcus aureus* by the TNO Microbiology and Systems Biology group (Montijn and Schuren personal communication). The final 512 compounds that showed to be active against *S*. *aureus* vegetative cells were selected for further analysis in this study.

We performed both population based assays and more detailed live imaging analyses on *B*. *subtilis* and *B*. *cereus* spores using SporeTrackerX. SporeTrackerX is software designed to evaluate the timing of germination and subsequent cell growth of bacterial spores from very large data sets. It runs under ImageJ in combination with the ObjectJ plugin. Like its predecessor “SporeTracker”^14^, it automatically locates spores that appear as bright objects in the first frame of a phase-contrast time-lapse sequence, then re-identifies and marks contours in subsequent frames and calculates parameters like germination time and generation time. For more information concerning SporeTrackerX, please refer to the supplementary material.

The SporeTrackerX analyses showed differences in sensitivity between *B*. *subtilis* and *B*. *cereus* spores that were not evident during the population based experiments, highlighting the importance that single cell live imaging can have in the screening of novel antimicrobials.

## Results

### Selection of five synthetic compounds showing inhibitory effects against B. subtilis spore outgrowth

**Figure 1:**
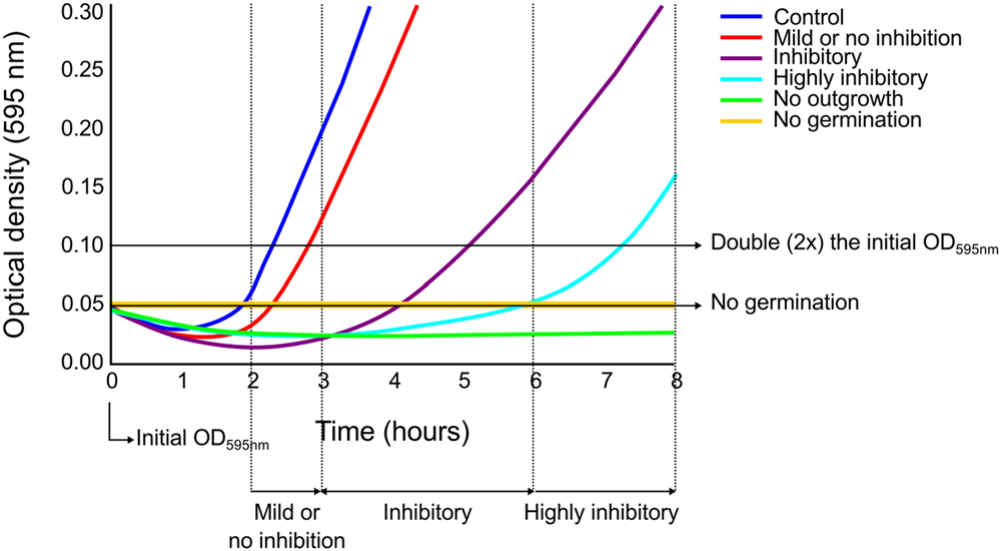
An illustration depicting the initial screening criteria. A benchmark line was set at two fold the initial optical density at an absorbance of 595 nm (OD_595nm_). Our operational definitions of inhibition by the antimicrobial compounds were as follows. ‘Mild or no inhibition’ was defined when the culture reached the benchmark line within 2 to 3 hrs. ‘Inhibitory’ was defined when the benchmark line was reached within 3 to 6 hrs and ‘highly inhibitory’ within 6 to 8 hrs. ‘No outgrowth’ was defined when no increase in OD_595nm_ was reached within 8 hrs. ‘No germination’ was demarcated when the OD_595nm_ did not lower the initial OD_595nm_ in time. Samples that did not show outgrowth after 8 hours were followed for maximally 48 hours.

**Figure 2:**
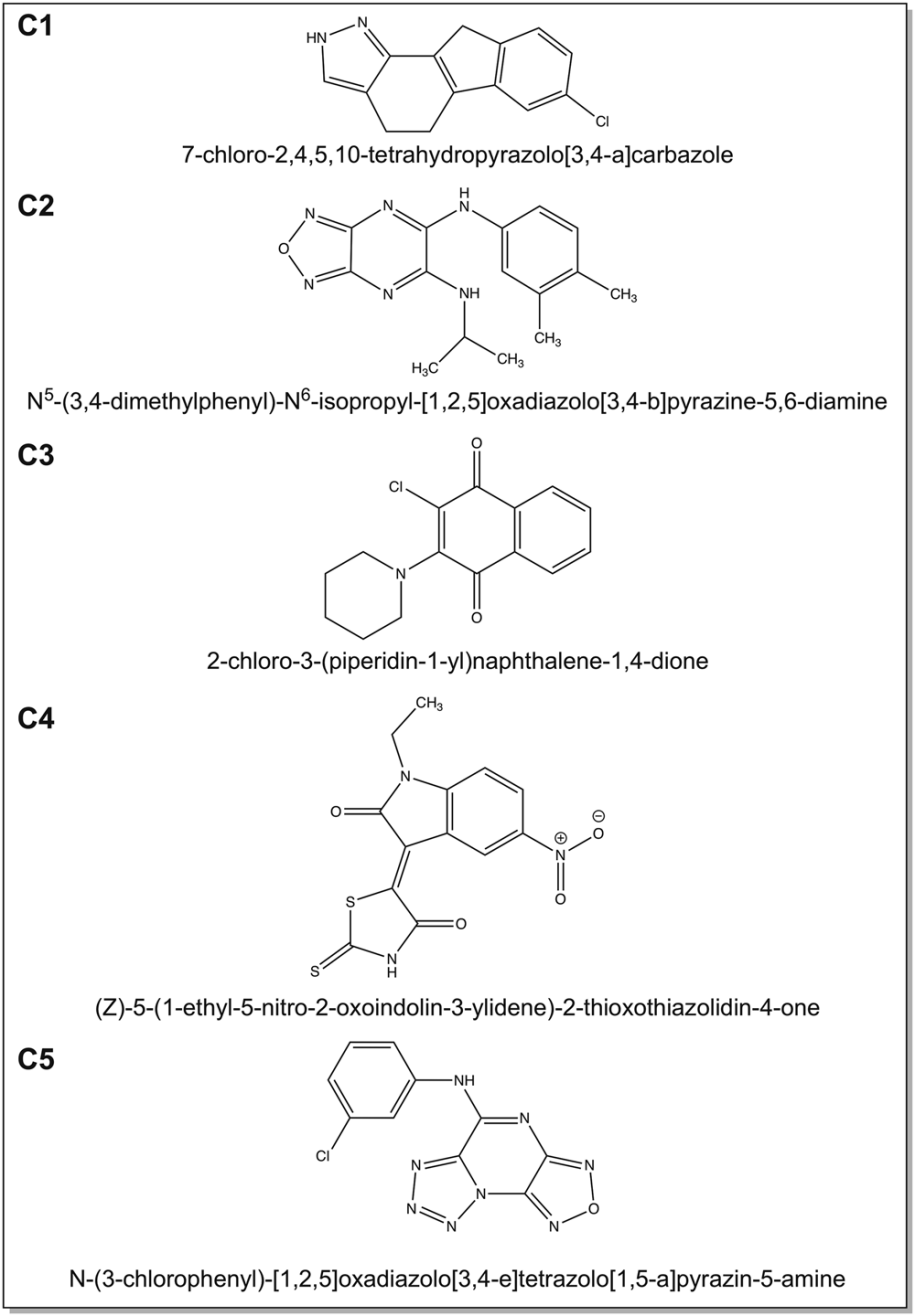
The names of the five compounds selected as determined with ChemDraw. The names for the compounds follows the International Union of Pure and Applied Chemistry (IUPAC) nomenclature.

The compound library from Pyxis Discovery B.V. (Delft, the Netherlands) enriched for activity against vegetative growth of *Staphylococcus aureus*, was initially screened against *B*. *subtilis* spores in a population based experiment where the optical density of the culture was observed for eight hours in the presence of 100 μg/ml of the compounds. Additional information about the 512 compounds can be found in the supplementary material together with the results of the initial screening. From this initial screen, fifteen compounds prevented spore germination, forty seven prevented outgrowth, and six compounds were highly inhibitory by preventing outgrowth for six hours (see for operational definitions of the level of inhibition in the initial screen Fig. 1 and the results reported in Table S1). The sixty eight compounds selected were subjected to an additional population based screen, which was performed in culture medium buffered at pH 7.4 or 5.9, at lower compound concentrations and for a longer treatment period of forty eight hours. We report here the data of the five most promising compounds that prevented outgrowth or germination, and could be re-synthesized at a scale sufficient for further experimentation (Table 1 and Fig. 2). The optical density curves of *B*. *subtilis* treated with the five compounds can be found in the supplementary figures.

**Table 1:** Synthetic compounds selected for inhibition of *Bacillus subtilis* spore germination or outgrowth.

Compound	pH 7.4, treated for 8 hrs		pH 5.9, treated for 8 hrs		pH 7.4, treated for 48 hrs		pH 5.9, treated for 48 hrs	
	Conc. (µg/ml)	Effect	Conc. (µg/ml)	Effect	Conc. (µg/ml)	Effect	Conc. (µg/ml)	Effect
C1	100	no germination						
	10	no outgrowth	10	no outgrowth	10	no outgrowth	10	no outgrowth
	1	mild/no inhibition						
C2	100	no outgrowth						
	10	inhibitory	10	no outgrowth	10	inhibitory	10	no outgrowth
	1	mild/no inhibition						
C3	100	no outgrowth						
	10	no outgrowth	10	no outgrowth	10	highly inhibitory for 10 hrs	10	highly inhibitory for 14 hrs
	1	mild/no inhibition						
C4	100	no germination						
	10	mild/no inhibition	10	mild/no inhibition		ND		ND
	1	mild/no inhibition						
C5	100	no outgrowth						
	10	no outgrowth	10	no outgrowth	10	no outgrowth	10	no outgrowth
	1	mild/no inhibition						

ND refers to not determined.

Compounds C1 and C5 prevented outgrowth of *B*. *subtilis* spores for 48 hours at pH 7.4 and 5.9. Compound C2 prevented outgrowth of *B*. *subtilis* spores for 48 hrs at pH 5.9, but was only inhibitory at pH 7.4. Compound C3, prevented spore outgrowth when *B*. *subtilis* was cultured at pH 7.4 and pH 5.9 for 8 hrs, and was considered “highly inhibitory” for 10 hrs at pH 7.4 and 14 hrs at pH 5.9. Compound C4 was the least promising compound, as it was only “mild or no inhibitory” at pH 5.9 for 8 hours. Still, both C4 and C1 were included in the subsequent experiments as the results of the initial screening suggested that C4 and C1 prevented germination at a concentration of 100 μg/ml in culturing medium buffered at pH 7.4 by preventing a decline in optical density for 8 hrs. These five compounds were further evaluated for minimal inhibitory concentration (MIC), minimal bactericidal concentration (MBC), and single spore live imaging.

### The minimal inhibitory concentration (MIC) and minimal bactericidal concentration (MBC) against *B*. *subtilis* and *B*. *cereus* spores

The MIC and MBC of the five synthetic compounds were determined to select concentrations for the subsequent single cell live imaging experiments. C1, C2, C3 and C4 were inhibitory against *B*. *subtilis* for 24 hrs at pH 7.5 (Table 2). C5, however, was inhibitory at a low concentration of 2 ± 1 µg/ml and bactericidal at a higher concentration of 9 ± 6 µg/ml against *B*. *subtilis* spores. C1, C3, and C4 were inhibitory against *B*. *cereus* spores, but C2 and C5 were bactericidal against *B*. *cereus* spores at 58 ± 42 and 213 ± 156 µg/ml, respectively. C5 was bactericidal at a concentration 23-fold higher against *B*. *cereus* than against *B*. *subtilis*.

**Table 2:** Minimal inhibitory concentration (MIC) and minimal bactericidal concentration (MBC) of the five synthetic compounds against *Bacillus subtilis* strain 168 and *Bacillus cereus* strain ATCC 14579. Data represent the mean of five biological repeats.

Article reference	*B*. *subtilis*		*B*. *cereus*	
	MIC (µg/ml)	MBC (µg/ml)	MIC (µg/ml)	MBC (µg/ml)
	*Mean* ± *SD*	*Mean* ± *SD*	*Mean* ± *SD*	*Mean* ± *SD*
C1	21 ± 17	<400	15 ± 7	<400
C2	20 ± 17	<400	10 ± 8	58 ± 42
C3	54 ± 39	<400	100 ± 40	<400
C4	75 ± 43	<400	30 ± 15	<400
C5	2 ± 1	9 ± 6	2 ± 1	213 ± 156

### Effect of compounds on individual *B*. *subtilis* and *B*. *cereus* spores

**Figure 3:**
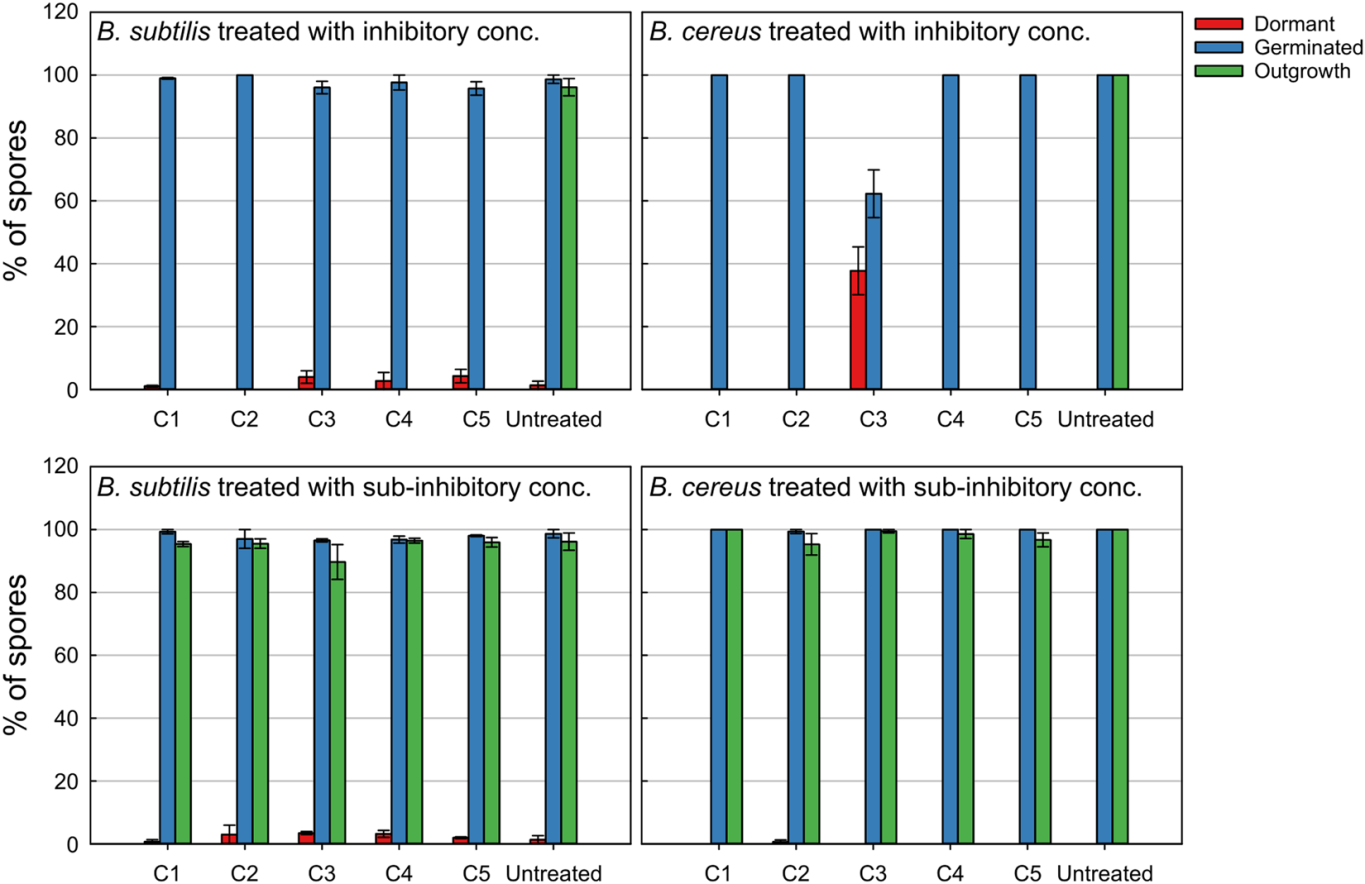
Quantification of the *B*. *subtilis* and *B*. *cereus* spores that germinated, grew out into vegetative cells or remained dormant from the total spores observed after 4.5 hrs of live imaging. The standard error bars represent two biological repeats. The number of spores assessed for each test condition can be found in Table 3.

Live imaging was employed to observe the antimicrobial effects of the selected synthetic compounds on *B*. *subtilis* and *B*. *cereus* spores at a single spore level. Two concentrations for each compound were selected, which were the minimal inhibitory concentration (MIC) and one sub-inhibitory concentration (sub-MIC), if not stated otherwise. The sub-MIC during the live imaging experiments was considered the highest concentration that still allowed spore outgrowth after 4.5 hours. The numbers of spores that remained dormant, germinated or grew out into vegetative cells were quantified (Fig. 3). The concentrations used can be found in Table 3. The five synthetic compounds prevented the outgrowth of germinated spores of both *B*. *subtilis* and *B*. *cereus* at inhibitory concentrations. More than 97% of the *B*. *subtilis* spores germinated after treatment with inhibitory concentrations of all compounds. Treatment with C1, C2, C4 and C5 lead to the germination of *B*. *cereus* spores, with no dormant spores remaining. Noteworthy was the spore germination inhibitory activity of C3 on *B*. *cereus* dormant spores. After C3 treatment, 38% (±22%) of *B*. *cereus* spores remained dormant for 4.5 hours and only 62% (±36%) germinated. The inhibitory activity of C3 on *B*. *cereus* spore germination was not observed for *B*. *subtilis*.

**Table 3:** Live imaging results after treatment of heat activated dormant spores with the synthetic compounds.

**Treatment**	Conc. (µg/ml)	Start of germination (min)				Germination duration (min)				Burst (min)				Generation time (min)			
***Bacillus subtilis***		*Median*	*p-value*	*IQR*	*Counts*	*Median*	*p-value*	*IQR*	*Counts*	*Median*	*p-value*	*IQR*	*Counts*	*Median*	*p-value*	*IQR*	*Counts*
Untreated	—	14	—	10	155	4	—	2	155	85	—	17	147	36	—	7	135
C1	21	17	≤0.01	19	188	3	≤0.01	1	188								
	5	14	0.65	10	112	2	0.28	2	112	131	≤0.01	38	102	55	≤0.01	12	90
C2	20	14	0.95	7	213	3	0.87	1	213								
	5	14	0.46	7	137	3	≤0.01	1	137	109	≤0.01	36	68	49	≤0.01	22	59
C3	54	15	0.09	13	223	3	≤0.01	1	223								
	3	14	0.86	9	210	3	≤0.01	2	210	104	≤0.01	26	189	49	≤0.01	17	177
C4	75	15	0.07	10	250	4	≤0.01	2	250								
	5	14	0.34	10	213	3	≤0.01	1	213	119	≤0.01	31	148	43	≤0.01	9	175
C5	6.4	14	0.18	12	250	3	≤0.01	2	250								
	1.6	14	0.71	7	212	3	≤0.01	1	212	99	≤0.01	24	199	38	≤0.01	3	187
***Bacillus cereus***																	
Untreated	—	4	—	1	118	1	—	1	118	75	—	14	111	37	—	3	101
C1	15	4	≤0.01	3	166	2	≤0.01	1	166								
	4	3	0.44	1	164	2	0.03	1	164	73	0.67	29	160	35	≤0.01	8	156
C2	10	3	≤0.01	1	265	2	≤0.01	1	265								
	1	3	≤0.01	2	130	2	≤0.01	2	130	83	≤0.01	25	103	38	≤0.01	9	87
C3	100	9	≤0.01	80	91	2	≤0.01	1	91								
	6	3	0.29	1	155	2	≤0.01	1	155	70	≤0.01	23	109	36	≤0.01	3	99
C4	30	3	≤0.01	1	224	3	≤0.01	2	224								
	4	3	≤0.01	1	107	2	≤0.01	1	107	116	≤0.01	36	93	43	≤0.01	7	84
C5	1.8	4	≤0.01	1	192	2	≤0.01	1	192								
	0.9	4	0.19	4	192	2	≤0.01	1	192	77	0.23	35	98	43	≤0.01	9	77

IQR refers to inter-quartile range.

**Figure 4:**
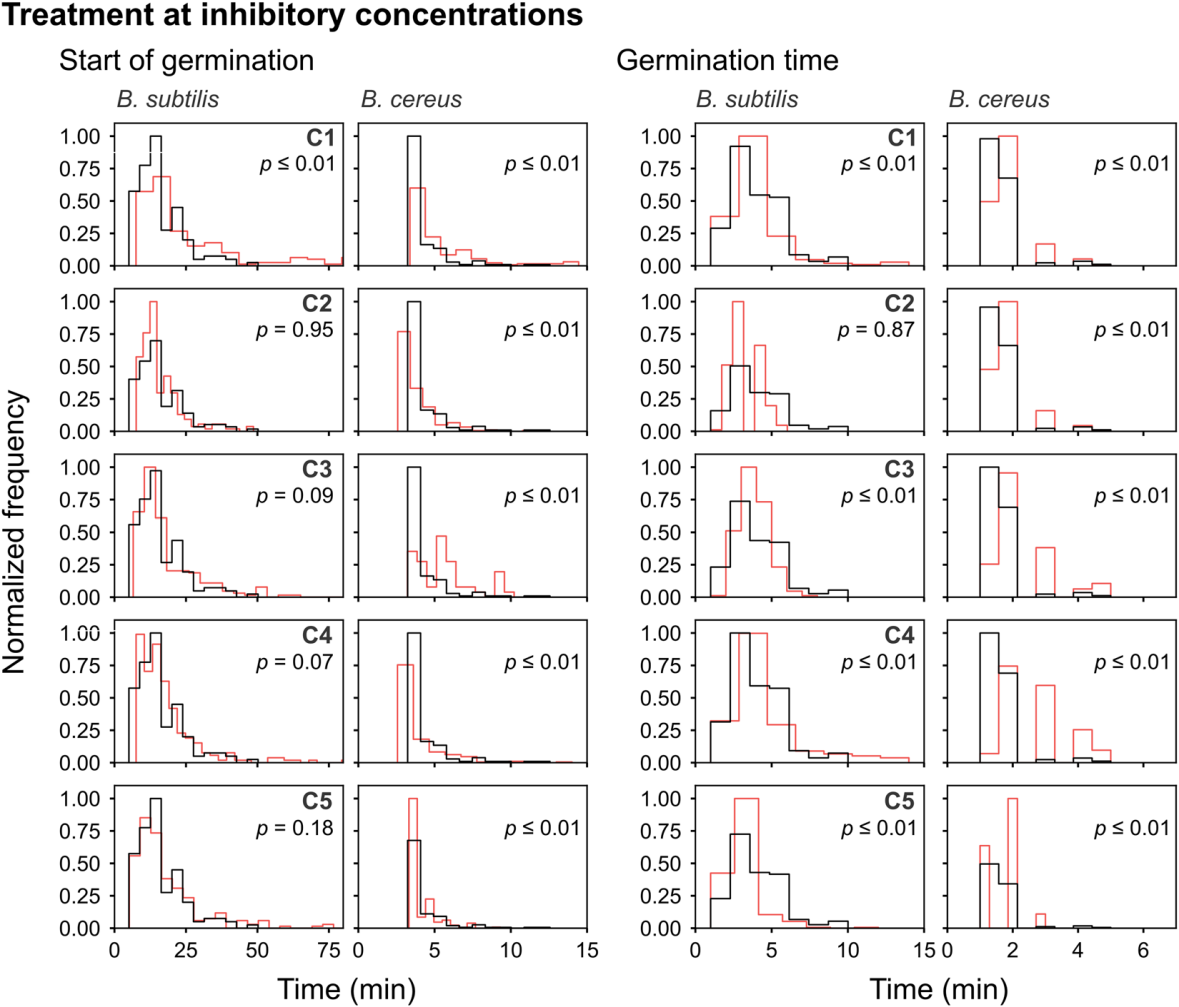
Frequency distribution curves of *B*. *subtilis* and *B*. *cereus* spores treated at inhibitory concentrations of C1, C2, C3, C4 and C5 (red line). The start of germination and germination time are depicted. Treated conditions were overlaid with untreated *B*. *subtilis* and *B*. *cereus* spores (black line). The histogram was normalized to occupy an area of one and was rescaled so that the maximum value in the histogram is equal to one. Significance of differences between the median values of the two groups was assessed using the Mann-Whitney test. Observations of two biological repeats were grouped and analysed as one data set. See Table 3 for the concentrations used.

**Figure 5:**
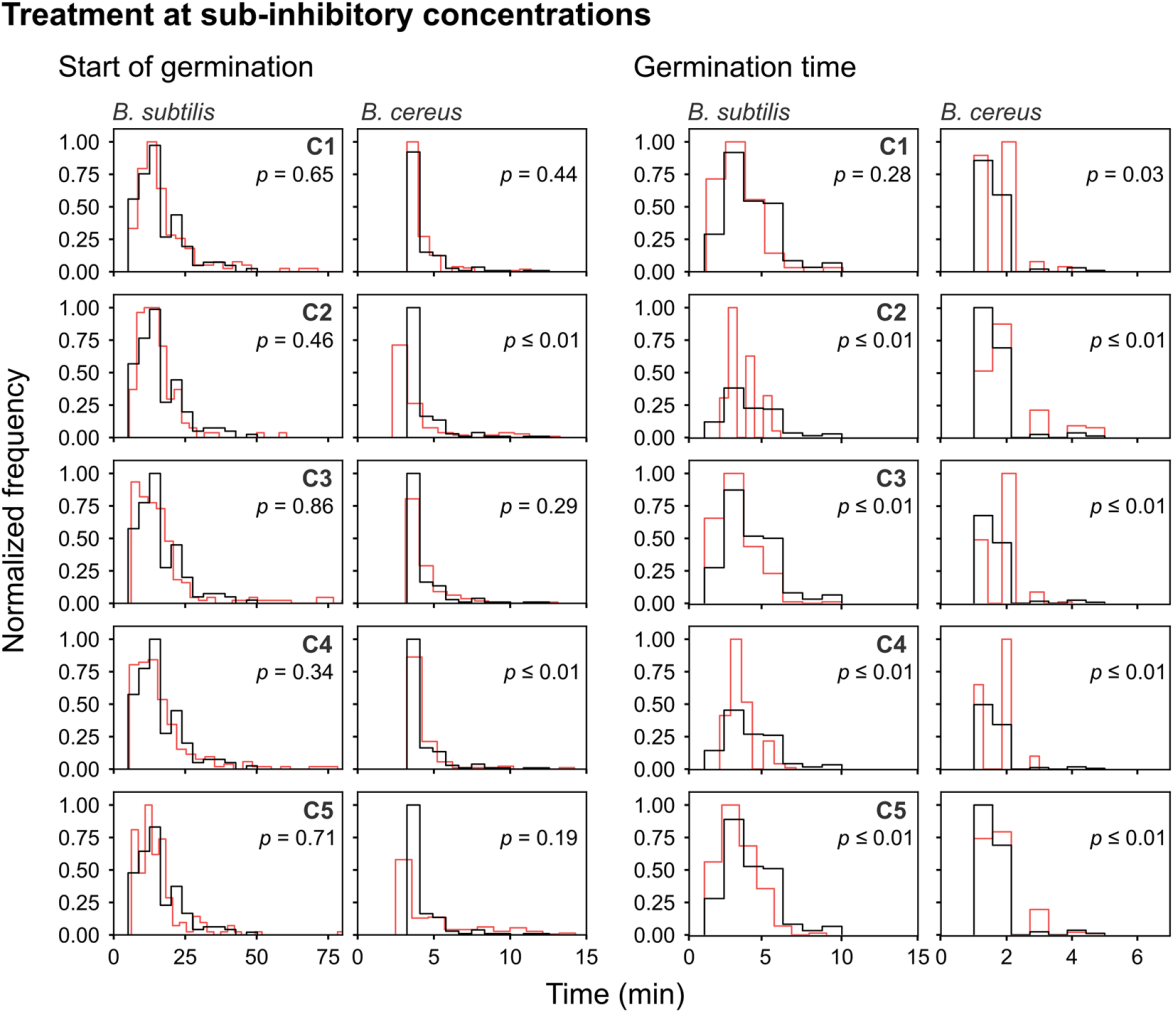
Frequency distribution curves of *B*. *subtilis* and *B*. *cereus* spores treated at sub-inhibitory concentrations of C1, C2, C3, C4 and C5 (red line). The start of germination and germination time are depicted. Treated conditions were overlaid with the untreated *B*. *subtilis* and *B*. *cereus* spores (black line). The histogram was normalized to occupy an area of one and was rescaled so that the maximum value in the histogram is equal to one. Significance of differences between the median values of the two groups was assessed using the Mann-Whitney test. Observations of two biological repeats were grouped and analysed as one data set. See Table 3 for the concentrations used.

**Figure 6:**
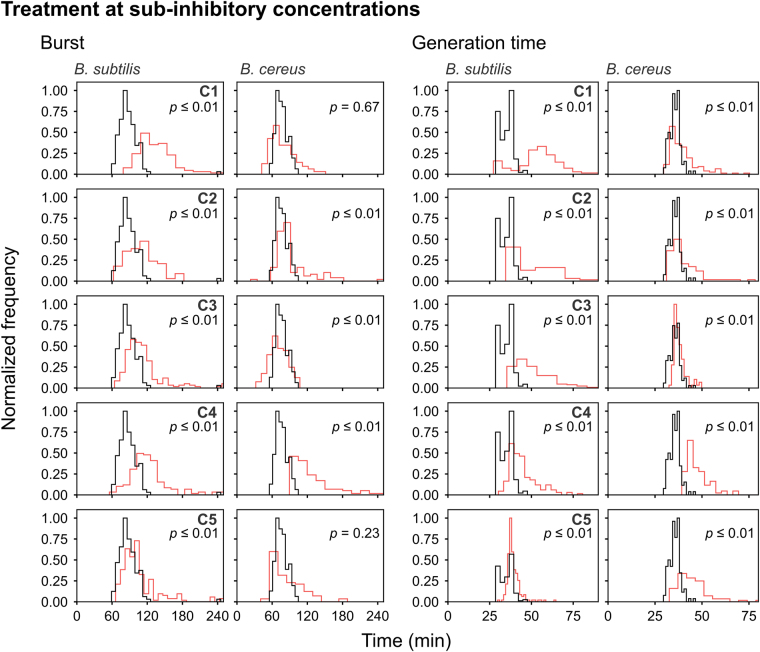
Frequency distribution curves of *B*. *subtilis* and *B*. *cereus* spores treated at sub-inhibitory concentrations of C1, C2, C3, C4 and C5 (red line). The burst and generation time are depicted. Treated conditions were overlaid with the untreated *B*. *subtilis* and *B*. *cereus* spores (black line). The histogram was normalized to occupy an area of one and was rescaled so that the maximum value in the histogram is equal to one. Significance of differences between the median values of the two groups was assessed using the Mann-Whitney test. Observations of two biological repeats were grouped and analysed as one data set. See Table 3 for the concentrations used.

In contrast to the MIC and MBC observations (Table 2), C5 appeared to be more active against *B*. *cereus* on solid medium than *B*. *subtilis* during the observation period. Outgrowth of *B*. *cereus* spores was inhibited by 1.8 µg/ml C5 whereas *B*. *subtilis* spores required a four-fold higher concentration of 6.4 µg/ml C5 (Table 3). The live imaging data were skewed to the right as can be observed from the frequency distribution curves in Figs 4, 5 and 6. In accordance with this, the data failed the Shapiro Wilk normality test. Thus, in order to analyse our results, we applied the Mann-Whitney test to determine *p*-values and probe for significance. The median values of the start of germination, germination time and burst time for untreated *B*. *cereus* was significantly (*p* ≤ 0.01) shorter than for *B*. *subtilis* (Table 3, Figs 4 and 5). The generation time was similar (*p* = 0.81) (Fig. 6). Therefore, *B*. *subtilis* and *B*. *cereus* only differed in their germination process in our test conditions. The data after analysing the live imaging movies with SporeTrackerX, can be found in the supplementary table (Tables S2 and S3).

The live imaging results showed that the inhibitory concentrations of C1 delayed the start of germination of *B*. *subtilis* spores with a difference in median value of 3 min, and a minor difference (<1 min) in the start of germination of *B*. *cereus* spores (Table 3 and Fig. 4). C2, C4 and C5 treated *B*. *subtilis* and *B*. *cereus* spores had a difference in median start of germination of ≤1 min, which suggest that their effect on this process is minor. C3, however, significantly delayed the start of germination of *B*. *cereus* spores with a difference in median value of 5 min. The results with *B*. *subtilis* spores treated with C3 were not as extreme, displaying a difference in median value of 1 min. The effect of inhibitory concentrations of the compounds on the germination time was negligible (<1 min).

At sub-MIC concentrations more than 99% of *B*. *cereus* spores germinated and 94% of the germinated spores grew out into vegetative cells (Fig. 3). *B*. *subtilis* spores, however, had ≤4% dormant spores still present after treatment with all five compounds. The effects of the compounds on the start of germination and germination time at sub-MIC concentration were negligible (≤1 min) (Table 3 and Fig. 5). The burst time of *B*. *subtilis* spores, however, was significantly affected by compounds C1, C2, C3, C4 and C5 with differences in median value of 46 min, 24 min, 19 min, 34 min and 14 min, respectively (Table 3 and Fig. 6). C1 and C3 shortened the burst time of *B*. *cereus* spores by causing the release of the spore-coat to occur earlier with a median value difference of 2 min and 5 min, respectively. C2, C4 and C5 significant delayed the burst time of *B*. *cereus* spores with a median value of 8 min, 41 min and 2 min, respectively.

C1, C2, C3, C4 and C5 significantly delayed the generation time of outgrowing *B*. *subtilis* vegetative cells with differences in median values of 19 min, 13 min, 13 min, 7 min and 2 min, respectively. C1, C2, C4 and C5 delayed the generation time of *B*. *cereus* cells with a median value difference of 2 min, 1 min, 6 min and 6 min. The generation time of *B*. *cereus* cells treated with C3 was increased by a minor median value difference of 1 min. In conclusion, both the burst time of *B*. *subtilis* and *B*. *cereus* germinated spores and the generation time of the vegetative cells were affected by the presence of the selected compounds.

### Correlations between germination and outgrowth phases, and mechanistic considerations

**Figure 7:**
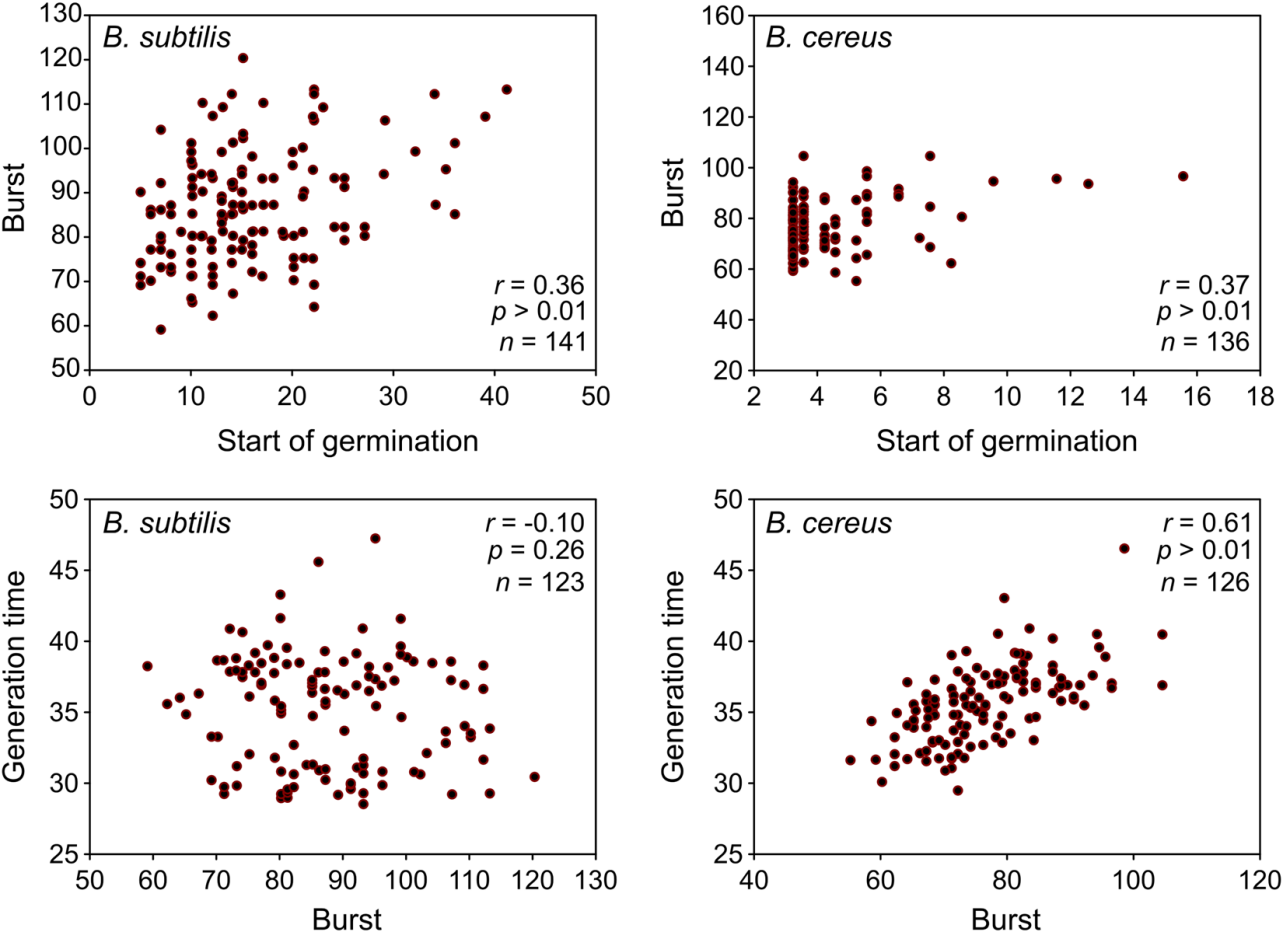
Scatterplots evaluating the correlation between the start of germination, bursting time and generation time of untreated *B*. *subtilis* and *B*. *cereus* spores. The Pearson’s correlation coefficient (*r*) was determined to estimate the strength of the relation. The *p*-value (*p*) for the correlation and the number (*n*) of comparisons are also shown. The start of germination was compared with the burst time, and the burst time with the generation time. The results showed that there was no correlation between the compared data sets. Observations of two biological repeats were grouped and analysed as one data set.

To assess whether the spores that germinate earlier will also burst earlier, the Pearson’s correlation coefficient was determined for both *B*. *subtilis* and *B*. *cereus* untreated spores. The start of germination for both *B*. *subtilis* and *B*. *cereus* did not correlate with the burst time, with the correlation coefficient (*r*) at 0.36 and 0.37, and with a *p*-value of > 0.01 and 0.26, respectively (Fig. 7). Similarly, the burst time did not correlate with the generation time (Fig. 7).

**Figure 8:**
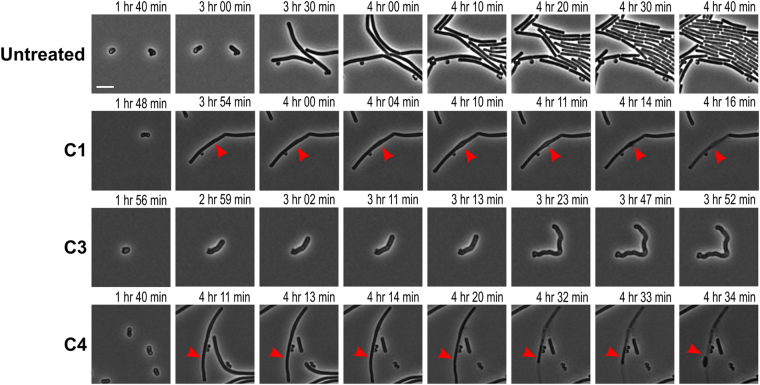
Snapshots of the live imaging results of *B*. *subtilis* spores untreated and treated with sub-inhibitory concentrations of C1, C3 and C4. Absolute time of the live cell imaging are shown from the start of sample preparation. Treatment with C1 show the start of cell wall damage 3 hrs and 54 min after the start of measuring, but cell lyse only occur 2.5 hrs after spore bursting (red arrows). Treatment with C3 resulted in enlarged cells that appeared to be swelling directly after bursting. The deformed cells appear to not divide, but elongate into a cell with irregular curvatures. Treatment with C4 also resulted in cell lysis 2.5 hrs after spore bursting (red arrows). Scale bar represent 2 µm.

We observed cell lysis about 2.5 hrs after bursting occurred of *B*. *subtilis* spores when treated with sub-inhibitory concentrations of C4 (Fig. 8). This observation suggests that the compound might be targeting the cell wall synthesis pathway or trigger autolysis. C1 also caused cell lysis of *B*. *subtilis* spores after 2.5 hrs of treatment with sub-inhibitory concentrations. The effect of C1 on the cell wall started with a bulging of the cell after 2 hrs and 17 min, which quickly leads to the lysis of the cell after 12 min, suggesting weakening of the cell wall. These findings were not observed with *B*. *cereus* spores. Finally, at sub-inhibitory concentrations of C3, outgrowing vegetative cells had visible morphological changes compared to untreated cells and to cells treated with the other compounds (Fig. 8). The cell width appeared wider, no visible cell division occurred and the elongating cells curled due to the irregular cell morphology. To obtain an overview of the irregular morphogenesis that the compounds induce we refer to the supplementary movies.

## Discussion

The data show that the definitions used in the initial rough screen for germination and outgrowth inhibition are not one-to-one comparable with the actually observed germination and outgrowth phenomena using our live imaging system. Differences include the way of assessing the effects, in solution or in a solid matrix, and the spore density being high or low. Low spore numbers are likely to be closer to reality than the high ones, and indeed growth on surfaces is more common in practice than growth in solution. Thus while the rapid characterisation of compounds using optical density is appropriate for fast characterisation, analysis using live imaging is crucial for detailed and more relevant investigations. During the analysis of our live imaging data we observed that the data is skewed and, therefore, failed the Shapiro-Wilk normality test. We expect the heat activation of our dormant spores and the use of nutrient-rich media will initiate rapid and relatively homogeneous germination. Hence the majority of the spores will germinate synchronously, while a small percentage remain dormant and germinate at a later stage^14–16^. The absence of correlations between the start of germination, burst time and the generation time is in agreement with the notion that all processes represent distinct biochemical events. That is, the interaction and activation of a pre-existing germination machinery, the initial phases of protein synthesis leading on to spore burst, and vegetative growth and cell division.

The five compounds analysed all showed future application potential as they had an inhibitory effect on the outgrowth of both *B*. *subtilis* and *B*. *cereus* germinated spores. The germination process of *B*. *subtilis* and *B*. *cereus* was unaffected by the presence of the compounds, except in the case of C3, which is similar to the observations made with sorbic acid^16^ and tea compound^15^ treatments of *B*. *subtilis*. Compound C2, N^5^-(3.4-dimethylphenyl)-N^6^-isopropyl-[1,2,5]oxadiazolo[3,4-b]pyrazine-5,6-diamine, and compound C5, N-(3-chlorophenyl)-‘[1,2,5]oxadiazolo[3,4-e]tetrazolo[1,5-a]pyrazine-5-amine were the only compounds that showed bactericidal activity. Compound C5 was bactericidal against both *B*. *subtilis* and *B*. *cereus* spores, whereas compound C2 was only bactericidal against *B*. *subtilis* spores. C5 during the initial screening proved to be active by preventing outgrowth for 48 hrs at both pH 7.4 and 5.9. C2 performed better at pH 5.9 by preventing outgrowth for 48 hrs and was only ‘inhibitory’, according to the definition of Fig. 1, at pH 7.4. C2 and C5 have the common features of containing heterocyclic oxadiazolo and pyrazine in their chemical structure. Compounds containing oxadiazolo^17–19^ or pyrazine^20^ are known for their antimicrobial activity. Interestingly, pyrazine was also shown to be a key feature of compounds produced by a soil bacterium *Paenibacillus* sp. that inhibits the growth of the Gram-negative *Burkholderia* sp.^21^, and by a endophytic *Bacillus megaterium* strain that inhibits the growth of various plant pathogens^22^.

Even though all five compounds delayed the bursting time of germinated *B*. *subtilis* spores at sub-MICs, compound C1, 7-chloro-2,4,5,10-tetrahydropyrazolo[3,4-a]carbazole, and compound C4, (Z)-5-(1-ethyl-5-nitro-2-oxoindolin-3-ylidene)-2-thioxothiazolidin-4-one, showed the most potential by delaying the process for longer than the rest of the compounds. These findings suggest that the compounds, especially C1 and C4, might form leads for application as complementary antimicrobials with other compounds that are slow acting on germinated spores. Delaying the burst time will delay the outgrowth of toxin producing vegetative cells and provide additional time for slow acting compounds to target the now more vulnerable germinated spore. The compounds were not as active in delaying the burst time of *B*. *cereus* spores, except for C4 that delayed the bursting significantly. Compound C1 contained pyrazolo[3,4-a]carbazole in its structure which are known for their antimicrobial activity against Gram-positives, -negatives and fungi^23^. C1 prevented outgrowth at pH 7.4 and pH 5.9 for 48 hrs. Compound C4 contained heterocyclic 2-thioxothiazolidin-4-one in its structure, which is another heterocyclic structure associated with antimicrobial compounds^24^. Interestingly, 2-thioxothiazolidin-4-one containing compounds have been shown to inhibit the MurD ligase involved in cell wall synthesis of *Escherichia coli*^25^. C1 and C4 were also selected for their putative spore germination inhibitory effect. However, under the live imaging conditions neither of these two compounds proved to be able to significantly inhibit the phase bright to phase dark transition, compared to the control incubation. Instead compound C3, 2-chloro-3-(piperidin-1-yl)naphthalene-1,4-dione, prevented spore germination of 38% of all *B*. *cereus* spores examined, and delayed the bursting of both *B*. *subtilis* and *B*. *cereus* spores at inhibitory concentrations. C3 was associated with the emergence of irregular cell morphology (Fig. 8). C3 has shown to be antifungal and antibacterial^26^. It is characterised by the heterocyclic structure, naphthalene-1,4-dione, commonly associated with natural occurring compounds such as phylloquinone and menaquinone (Vitamin K), antitumor drugs daunorubicin and doxorubicin produced by *Streptomyces*^27^, RNA polymerase inhibitor myxopyronin produced by soil bacterium *Myxococcus fulvus*^28^, naphthazarin produced by *Fusarium solani*^29^, and 2-hydroxy-1,4-naphthoquinone (lawsone or henna)^30^. Naphthalene-1,4-dione containing compounds have been associated with antimycobacterial^31^, and more generic antimicrobial activities^32^. What makes C3 the most promising candidate for further analysis is its ability to prevent germination and delay of outgrowth of spores, which might have an important application in the food chain where *B*. *cereus* spores are a main concern. *B*. *cereus* germinates rapidly, a phenomenon confirmed in our study, and grows out into vegetative cells where it gives the bacterium the advantage of dominating the environment. *B*. *cereus* are responsible for two types of food-borne illnesses; the emetic and diarrheal syndrome^4^. Emetic syndrome is associated with a dodecadepsipeptide toxin, celeulide, that is produced before the ingestion of contaminated food. Diarrheal syndrome occurs when living vegetative cells or spores are consumed^4,33^, and survive the acidity of the human stomach depending on the food^34^. Growing vegetative cells in the gut can produce sufficient amounts of enterotoxins, hemolysin BL (HBL), non-haemolytic enterotoxin (NHE) or cytotoxin K, causing abdominal pain followed by watery diarrhoea, and sometimes nausea and vomiting^4,33^. Even though the actual implementation of a compound for clinical use requires extensive research that is beyond the scope of the study, it is tempting to speculate that C3 (or similar naphthalene-1,4-dione containing compounds) might be useful in preventing spore germination in the food chain thus aiding in the prevention of the diarrheal syndrome. Insight in the stage at which spore germination is perturbed upon incubation with C3 will provide a mechanistic basis for its functioning. A recent review by Setlow *et al*.^35^ discusses current mechanistic knowledge of spores and spore germination. While *B*. *cereus* germination progresses mechanistically along analogous lines as we know for *B*. *subtilis*, the actual germinants are different and a germinosome, cluster of germination proteins, is yet to be uncovered in *B*. *cereus*. Finally, the chemical structures identified in the current study can be used as input to screen natural compound libraries to identify suitable natural equivalents with potentially equal potency^36^.

## Conclusion

In our study we employed an empirical approach to search for novel antimicrobial compounds active against Gram-positive spore-forming bacteria. We selected five compounds that showed potential as antimicrobials with possible different modes of action against *B*. *subtilis* and *B*. *cereus* spores. During the MIC and MBC, the five compounds did not show dramatic differences in their activity against *B*. *subtilis* and *B*. *cereus* spores, however, the live imaging analysis highlighted key differences in activity against the two bacteria, for instance in the case of C3. These findings stress that the choice of an appropriate model microorganism used during the screening of compounds is essential in identifying novel potent compounds, but also highlights the importance of single cell analysis in the screening for novel antimicrobial compounds.

## Materials and Methods

### Synthetic compounds used

The compounds used in the study were obtained from Pyxis Discovery B.V. (Delft, the Netherlands). Compounds were dissolved in diethyl sulfoxide (DMSO) (≥99.9% purity, Sigma-Aldrich) to a final concentration of 5 mg/ml. The approach used for the selection of the compounds made use of the principles described by Siegal, G., AB, E. & Schultz^8^.

### Preparation of *B*. *subtilis* and *B*. *cereus* spores

For the preparation of *B*. *subtilis* strain 168 and *B*. *cereus* strain ATCC 14579 spores, the method described by Abhyankar *et al*.^37^ was followed. In short, a single colony of *B*. *subtilis* or *B*. *cereus* from tryptic soy broth (TSB) solid medium was inoculated in 5 ml TSB liquid medium and incubated overnight at 37 °C while shaking at 200 rpm. The overnight culture was re-inoculated into fresh 5 ml TSB and cultured until an optical density, at an absorbance of 600 nm (OD_600_), of 0.3 to 0.4 was reached. This step ensured that the culture used was in the exponential growth phase. A serial dilution of the culture was performed in a defined minimal medium for *B*. *subtilis* to condition the cells to the medium used in the subsequent steps and to keep them in exponential phase. The defined minimal liquid medium was buffered with 3-morpholinopropane-1-sulfonic acid (MOPS) to pH 7.4 and supplemented with 10 mM glucose and 10 mM NH_4_Cl. For *B*. *cereus*, a serial dilution was performed in chemically defined growth and sporulation (CDGS) medium^38^. A dilution with an OD_600_ of 0.3 to 0.4 was selected and 1 ml of this dilution was inoculated in 20 ml pre-warmed medium until an OD_600_ of 0.3 to 0.4 was reached. Finally, 2.5 ml of the culture was inoculated in 500 ml pre-warmed medium and incubation for 72 hours at 37 °C for *B*. *subtilis* and 120 hours at 30 °C for *B*. *cereus*, while shaking. When >95% spores were obtained, the spores were pelleted at 4256 RCF for 15 min at 4 °C and the supernatant discarded. The spores were washed thrice and re-suspended with pre-chilled sterile MilliQ water. Residual vegetative cells and germinated spores were removed using Histodenz (Sigma-Aldrich) as described in^39^. Prior to treatment with the synthetic compounds, *B*. *subtilis* dormant spores were heat activated for 30 minutes and *B*. *cereus* dormant spore for 15 minutes at 70 °C. Culturing in all subsequent steps was at 37 °C for *B*. *subtilis* and 30 °C for *B*. *cereus*.

### Primary screening for activity of synthetic compounds against *B*. *subtilis* spores

Screening of the synthetic compounds was performed in liquid medium containing TSB, buffered with 80 mM MOPS to pH 7.4 or 80 mM 2-(N-morpholino)ethanesulfonic acid (MES) at pH 5.9. The spore germinants AGFK (10 mM L-asparagine, 10 mM glucose, 1 mM fructose and 1 mM potassium chloride) were included in the culturing medium to ensure optimal germination conditions. Heat activated spores were added to have a final optical density at a wavelength of 595 nm (OD_595_) of 0.2 (1 × 10^8^ CFU/ml). The effects of the synthetic compounds were assessed by measuring the OD_595_ at 37 °C while shaking using a microtiter plate reader (Multiskan FC, Thermo Scientific). Compounds were selected for their prevention of spore germination or outgrowth initially at 100 µg/ml during a time-frame of 8 hrs at pH 7.4, followed by a time-frame of 48 hrs at pH 7.4 or pH 5.9 and at final concentrations of 1 μg/ml, 10 μg/ml or 100 μg/ml.

### Determination of the MIC and MBC

To obtain the lowest concentration necessary to have an inhibitory effect on *B*. *subtilis* and *B*. *cereus* spores, the MIC was determined. The MBC was determined to establish whether the synthetic compounds are lethal at a concentration close to the MIC. This was performed by measuring the OD_595_ for 24 hours in a microtiter plate reader (Multiskan FC, Thermo Scientific). The culture was incubated with a final OD_595_ of 0.2 in liquid medium containing TSB, buffered with MOPS at pH 7.4, containing the compounds. A two-fold serial dilution from 400 µg/ml to 0.40 µg/ml of the synthetic compounds was prepared. The control consisted of TSB, buffered with MOPS at pH 7.4, without the compounds. An additional control was included, where the medium contained DMSO at the maximum volume added of the compounds under study. The experimental conditions to determine the MBC were similar to the MIC, but after 24 hours the culture was plated out onto TSB solid medium. The MIC was considered to be the concentration where no outgrowth (increase in OD_600_ over time) was observed and the MBC where 99.99% of the culture produced no CFU after 24 hrs. For the culturing of *B*. *subtilis* the culturing medium contained the germinants AGFK while for *B*. *cereus* 10 mM inosine was included. Biological repeats were performed.

### Live imaging of spores to observe the antimicrobial effect of the synthetic compounds

Heat activated spores were observed over time when treated with the synthetic compounds in two different test conditions: (1) Synthetic compounds were added at sub-inhibitory concentrations (outgrowth occurs) and (2) Inhibitory concentrations (that prevented outgrowth) were present in the solid culture medium during imaging. The exact concentrations of each compound tested can be found in Table 3. The solid culture medium contained TSB and 1% agarose. The germinants AGFK were additionally included in the culture medium for *B*. *subtilis*, while for *B*. *cereus* the germinant inosine was added. Microscope slide preparation and imaging was performed as described by Pandey *et al*.^14^. Microscopy images were analysed in ImageJ (http://rbsweb.nih.gov/ij/). Live imaging was performed with the Nikon Eclipse Ti. The Nikon Eclipse Ti had for phase contrast imaging a Prior Brightfield LED, a Nikon CFI Plan Apo Lambda 100X Oil, C11440‐22CU Hamamatsu ORCA flash 4.0 camera, LAMBDA 10-B Smart Shutter from Sutter Instrument, an OkoLab stage incubator and was equipped with NIS elements software version 4.50.00. The start of germination, germination time, and burst of each spore as well as the generation time of the outgrowing vegetative cell were assessed with the aid of the ImageJ plugin SporeTrackerX designed by Norbert Vischer (see supplementary material and https://sils.fnwi.uva.nl/bcb/objectj/examples/sporetrackerx/MD/sporetrackerx.html). All statistical analysis was performed in SigmaPlot 13.0 (Systat Software Inc.). The start of germination is the beginning of the transition from phase bright spores to phase dark spores, and the end of germination is when this transition comes to an end^14,16^. The germination time is the difference in time between the start and end of germination. The burst time is the time when the spore-coat is shed. The generation time is the area of a cell or colony over time^14,16^.

### Data availability

The datasets generated during and/or analysed during the current study are available from the corresponding author on request.

## Electronic supplementary material

 Supplementary Information

S1Table_List of 512 compounds screened

S2Table_Bsubtilis_LiveImaging_Dataset

S3Table_Bcereus_LiveImaging_Dataset

SFigures_Growth curves of B. subtilis treated with the five compounds

SMovie2_Bsub_C1Treated

SMovie4_Bsub_C4Treated

SMovie1_Bsub_Untreated

SMovie3_Bsub_C3Treated
