# Supplementary material for: Evaluating novel synthetic compounds active against Bacillus subtilis and Bacillus cereus spores using Live imaging with SporeTrackerX
Source: Sci Rep. 2018 Jun 14;8:9128. doi: 10.1038/s41598-018-27529-4 (PMC6002552; doi:10.1038/s41598-018-27529-4)
Supplement: Supplementary file 1 — Supplementary Information [file 41598_2018_27529_MOESM1_ESM.pdf]

Supplementary information

Evaluating novel synthetic compounds active against *Bacillus subtilis* and *Bacillus cereus* spores using Live imaging with SporeTrackerX

Soraya Omardien, Alexander Ter Beek, Norbert Vischer, Roy Montijn, Frank Schuren and Stanley Brul

Legends of supplementary movies

Movie S1. Live-imaging of untreated *Bacillus subtilis* spores for 4.5 hrs. Movies were constructed with 10 frames per second, where each frame represent 1 minute. The sample preparation time was 10 minutes for this movie.

Movie S2. Live-imaging of *Bacillus subtilis* spores treated with a sub-inhibitory concentration of C1 for 4.5 hrs. Movies were constructed with 10 frames per second, where each frame represent 1 minute. The sample preparation time was 8.5 minutes for this movie

Movie S3. Live-imaging of *Bacillus subtilis* spores treated with a sub-inhibitory concentration of C3 for 4.5 hrs. Movies were constructed with 10 frames per second, where each frame represent 1 minute. The sample preparation time was 6 minutes for this movie

Movie S4. Live-imaging of *Bacillus subtilis* spores treated with a sub-inhibitory concentration of C4 for 4.5 hrs. Movies were constructed with 10 frames per second, where each frame represent 1 minute. The sample preparation time was 6.5 minutes for this movie

SporeTrackerX technical description – scientific software for handling large live imaging data sets from bacterial spores

Abstract

Bacterial sporulation, followed by a heterogenous germination and outgrowth, is a strategy of bacteria to survive harsh environmental conditions. The examination of such heterogeneity is essential in the search for improved preservation methods in the food industry. To further analyse these processes and to obtain results with statistical significance, live imaging with a time resolution down to one minute has become instrumental. However, a complex experimental set-up can lead to the acquisition of hundreds of gigabytes of data. Here, we present the ImageJ-based software SporeTrackerX that optimises the speed of analysis and the handling of image data. After analysis it provides a navigation tool for instant real-time play-back of individual spores by using the corresponding growth and germination plot as a control panel. In this stage, the user can interactively overrule automatically detected events such as the spore burst or generation time of outgrowing vegetative cells. We also propose a naming scheme, file structure and a backup method to assist the analysis of image data close to the terabyte range. In conclusion, SporeTrackerX facilitates the analysis of data generated through live imaging, thus contributing to the understanding of the molecular physiology of spore-forming bacteria exposed to various environmental conditions.

1. Introduction

Bacteria of the order *Bacillales* and *Clostridiales* tend to survive harsh environmental conditions that might otherwise kill vegetative cells, by forming resilient dormant spores. These dormant spores withstand conditions such as high temperatures, radiation (UV and γ), toxic chemicals, desiccation, or freeze-thawing [1–3], but lose this resistance once the committed process of germination is initiated. Spores are the greatest concern in the food industry as they are a major cause of food-spoilage and food-borne illnesses, such as those produced by *Bacillus cereus*,

Clostridium perfringens or *Clostridium botulinum* [4]. Spores are also prevalent in health-care where *Clostridium difficile* is the greatest concern [5], and have been used for bioterrorism, such as the notorious *Bacillus anthracis* [6]. Not all spore-formers are harmful, for instance *Bacillus subtilis* with the generally regarded as safe (GRAS) status is commonly used as a model organism and has been proposed as a delivery system for vaccines, as a vaccine adjuvant [7], and as a probiotic [8,9].

As mentioned earlier, dormant spores loose their resilience once committed germination occurs. Therefore, effort is placed in understanding the germination process of spores. Spore germination has been discussed in-depth by Setlow et al. (2017) [10]. Spore-formers circumvent exposing the entire population to external threats through heterogeneous germination, which is not well understood. It requires only the survival of one dormant spore to continue a lineage and to cause, for instance, food-spoilage and food-borne illnesses. This phenomenon cannot be clarified using population based studies. As a consequence, focus is placed on single cell analyses. However, techniques such as flow cytometry or standard microscopy techniques are not sufficient. Therefore, live imaging of single cells has been used to observe the effects of various treatments that affect growth, spore morphogenesis and spore germination [11,12]. For live imaging of spores phase-contrast microscopy is used, where the uptake of water during the germination process changes the refraction index of light causing phase-bright dormant spores to appear as phase-dark germinated spores. Further imaging shows the bursting of each individual spore, the first division and the growth of vegetative cells. Often large scale experiments are needed, particularly when screening for compounds that interfere with spore germination. To convert the observation of each spore into numerical values, SporeTrackerX [https://sils.fnwi.uva.nl/bcb/objectj/examples/sporetrackerx/MD/sporetrackerx.html] was designed that enables semi-automated large scale image analysis of each stage from dormant spore to vegetative growth. SporeTrackerX runs in combination with ImageJ and plugin ObjectJ. Compared to its predecessor SporeTracker [11], it has many improvements as described below, including the optimisation to handle data sets of hundreds of gigabytes. In this paper, the design of SporeTrackerX will be discussed together with the various steps needed during its utilisation. *Bacillus cereus* and *Bacillus subtilis* spores will be used as an example for the SporeTrackerX image analysis.

2. SporeTrackerX as ObjectJ project

The popularity of the image analysis program ImageJ is greatly based on its open source character with the flexibility to add user-defined functionality via plugins. Among hundreds of plugins, several are specialized for the analysis of bacterial cells. Such programs can be very feature-rich and typically supply complex dialog boxes to cover as many as possible scenarios. However, due to their complexity and size, such plugins are not suitable for creating and maintaining personal branches in case the used architecture is not suitable for the researcher's problem. In our project, it was essential that the overlay markings were not saved together with the large movie stacks, but still kept consistently together for all movies of a population.

For SporeTrackerX, we use the plugin ObjectJ [https://sils.fnwi.uva.nl/bcb/objectj] rather than creating a new plugin. ObjectJ's unique feature of embedded macros provides an additional level of flexibility with easy user access. Local project files with the extension ".ojj" contain the embedded macro code together with other dynamically linked information, like non-destructive hierarchical markers, results and plot commands. A project file is saved next to the analysed image files in the same folder. Earlier projects, such as Coli-Inspector [13], ChainTracer [14] and NucTracer [14], have been realised successfully this way. SporeTrackerX is superior to its predecessor SporeTracker [11] due to the fully virtual concept that makes it suitable for data sets that reach the terabyte range, visualisation of colony contours as hierarchical objects, better navigation between plots and images, automatic handling of unstable movies without preprocessing, absence of auxiliary files, short file names with retrieval of the original names on demand, statistical isolation of different batches, and controlled interrupt for inspection during long processes. SporeTrackerX accepts multi-channel hyperstacks with phase contrast images in the first channel, but only single-channel experiments are described here.

Embedded macros use ImageJ's proprietary macro language which has been extended for ObjectJ's ability to dynamically link all acquired information across many images or movies. Compared to the two other supported interpreted languages, JavaScript and Python, it is simpler, not object-oriented, and pragmatically tailored to directly interact with ImageJ's features. It provides a single-step debugger and can be handled or modified by users with minimal programming skills. Embedded macros in ObjectJ projects typically focus on special tasks without packing more functions or dialog boxes than necessary into the project file. For example,

we do not anticipate all possible thresholding methods, instead we rely on the fact that the code can be easily fine-tuned in a responsive, flexible and well-documented manner while no separate source or setting files need to be maintained.

This way, both the data and the functionality are contained in one small, manageable project file that does not depend on files other than the images. The macro language is typically used to handle the graphical user interface (GUI) and to organise the execution of calculation-intensive methods which run in native Java code at maximum speed.

3. Large volume experiments and virtual stacks

When experiments involve data towards the terabyte range, issues associated with storage and processing speed need to be carefully addressed. Operations that otherwise are trivial, like opening a file, creating duplicates, or performing a backup, can become serious bottlenecks during usage. For example, saving ImageJ's non-destructive overlay would typically be saved together with the image or stack itself which can be a gigabyte-operation for each file. In contrast, the plugin ObjectJ circumvents this problem as the overlays of all linked images are saved separately in one small project file, which in turn maintains the dynamic link between images, results and plots. Once the large timelapse files are in place, they are never re-saved.

ImageJ allows image stacks to be opened virtually. The use of virtual stacks allows the user to browse instantly through a part of a spore’s history without needing to load the entire movie in advance. A virtual stack can be larger than the computer's RAM, as only the currently visible image (or set of image channels) is kept in memory. SporeTrackerX utilizes virtual stacks by default and therefore will less likely be limited by computer hardware.

4. File organisation and naming scheme

Before acquiring hundreds of very large files on the microscope, we designed the file logistic in advance by creating an empty tree of directories using short and meaningful folder names (Fig. 1). Live imaging, in our case, was performed with a Nikon Eclipse Ti microscope. Its acquisition

software compiles many timelapse movies belonging to an experiment into a single large file with ".nd2" extension, each of which we stored in the correct position in the directory tree. However, for full flexibility in ImageJ, an extra conversion step from ".nd2" to ImageJ's preferred ".tif" format was necessary. For this, a separate utility macro "Import_ND2_files" [https://sils.fnwi.uva.nl/bcb/objectj/examples/Import_ND2_files/] was created which repeatedly calls the plugin BioFormats [https://www.openmicroscopy.org/bio-formats] to perform the conversions for the entire directory tree. SporeTrackerX does not use .nd2 files for analysis.

In lab practice, file names can become very long when experimental details are included as string fragments. Then they often are only partially visible in the graphical user interface and tend to make human communication error prone. We, therefore, decided to create short file names when resolving an .nd2 parent file into a series of individual .tif movies. For example in Fig. 1 column 3, the file name "Ser_328899_01.tif" was composed to hold a six-digit checksum ("328899") which was derived from the .nd2 parent name. The long name will then remain hidden in the TIFF metadata field. In Fig 1 column 4 it is immediately visible that the twelve .tif movies have two different parents, which in our case meant two different biological repeats ("batches"). SporeTrackeX runs under plugin ObjectJ, whose qualification mechanism allows to statistically isolate such batches to expose unexpected differences. The naming scheme with short names described here is optional, as SporeTrackerX accepts any file name.

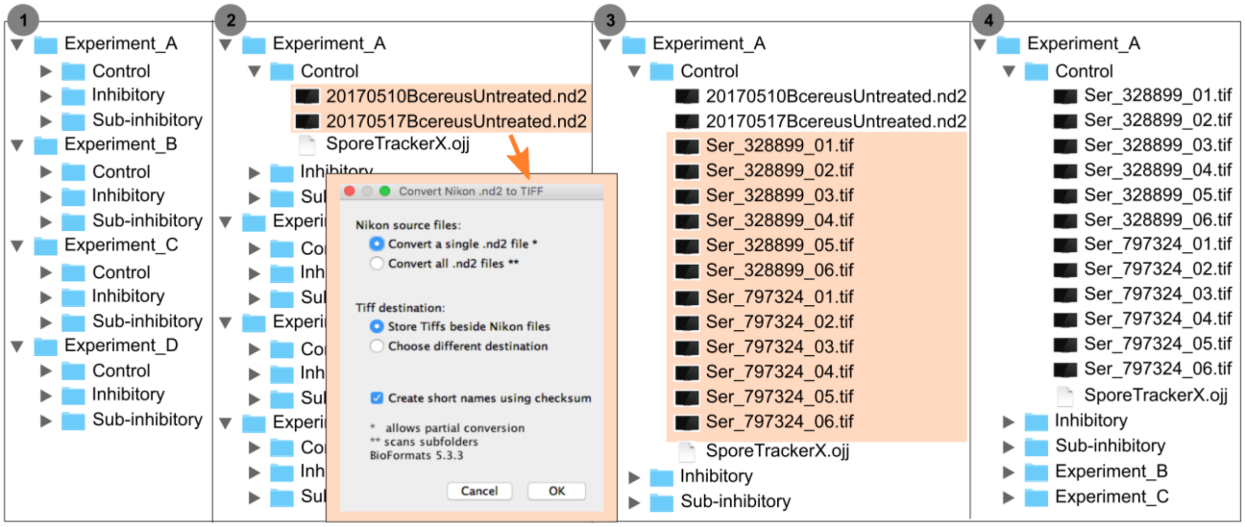

Figure 1. File organisation and naming scheme. In step 1, empty folders are created that represent the general workflow. In step 2, the directory tree is populated with Nikon ".nd2" movie sets. Additionally, each experiment obtains an empty copy of SporeTrackerX.ojj. In step 3, a utility macro called "Import_ND2_files" provides options (arrow) to convert each .nd2 file in the directory tree to a series of .tif files at the same location (as shown here), or to a different destination. Optionally, short file names with a 6-digit checksum are used to simplify user communication. In step 4, the .nd2 files can optionally be deleted.

5. Image Analysis

SporeTrackerX requires the plugin ObjectJ, which works with project files that have the extension ".ojj". A project file contains all image names, methods, data, plot commands and statistics that belong to an individual experiment. SporeTrackerX.ojj is such a project file specialized for the analysis described in this article. Before starting with the analysis of a cell population, an empty copy of the downloaded project file has to be stored in the directory holding the corresponding timelapse movies. These must then be linked to the project, which can be done with a drag-and-drop operation. When a project file is loaded, the ObjectJ menu becomes available in the main menu bar, showing a number of general ObjectJ commands in the upper part, and SporeTrackerX-specific embedded macro commands below these (Fig. 2A).

Embedded macro commands under the ObjectJ menu are arranged in the natural order they will typically be invoked in. The first of these commands is ObjectJ>Analyze Spores, which performs the major image analysis across all linked movies. As we work with virtual stacks, care is taken that a certain time frame is loaded only once from the disk into the memory, acquiring all desired information without the need to re-load it later.

Analysis is performed by running ImageJ's particle analyzer twice per frame to detect bright and dark objects. From the first frame, the range between background (modal value) and the maximum intensity is derived, and two thesholds at 40% ("bright") and 20% ("stillBright") of this range are defined. The particle analyzer then applies the 40% threshold and marks the found centroids in frame #1 with a red "Bright" marker as starting spore. However, close neighbors are

rejected. Continuing with frame #2, bright particles are accepted above the 20% level, and are marked as "Bright", unless a dark particle, first as a concentric ring, will appear at the same position. In the second run for the same frame, dark particles (outgrowing cells) are detected using ImageJ's "Default" threshold. They will, from now on, define the centroid and are marked with a green "Contour" marker. Objects are re-identified (traced) correctly if the frame-to-frame shift of the particle's controid is below the defined "jumpTolerance", otherwise the history will be closed. Abrupt changes in size or centroid position are interpreted as collision with a different colony and will stop further analysis. The measurements per spore and frame include intensity and area, both of which will be used to create the corresponding plot windows. 'Intensity' is sampled and averaged within a radius of 4 pixels from the centroid until a specified time after the bright-to-dark transition and within the entire particle's contour thereafter. 'Area' is the contour area of dark particles.

The plugin ObjectJ supports "composite objects". SporeTrackerX makes use of composite objects by packing the information of the entire life time of a spore into a single object, including "Bright" and "Contour" markers across many stack frames. Each spore’s history is connected to a single row in the ObjectJ results table, subdivided in various columns for strings or numbers. Also arrays holding the profiles of intensity and area versus time are stored in special result columns as string constructs and can be retrieved for reconstruction of the correponding plots. Calculation of the start and end of germination, detection of the time point of the shedding of the spore coat (burst), and calculation of the generation time are described in the first version of SporeTracker [11]. All these results become visible in the ObjectJ results table (Fig. 3), which is dynamically updated.

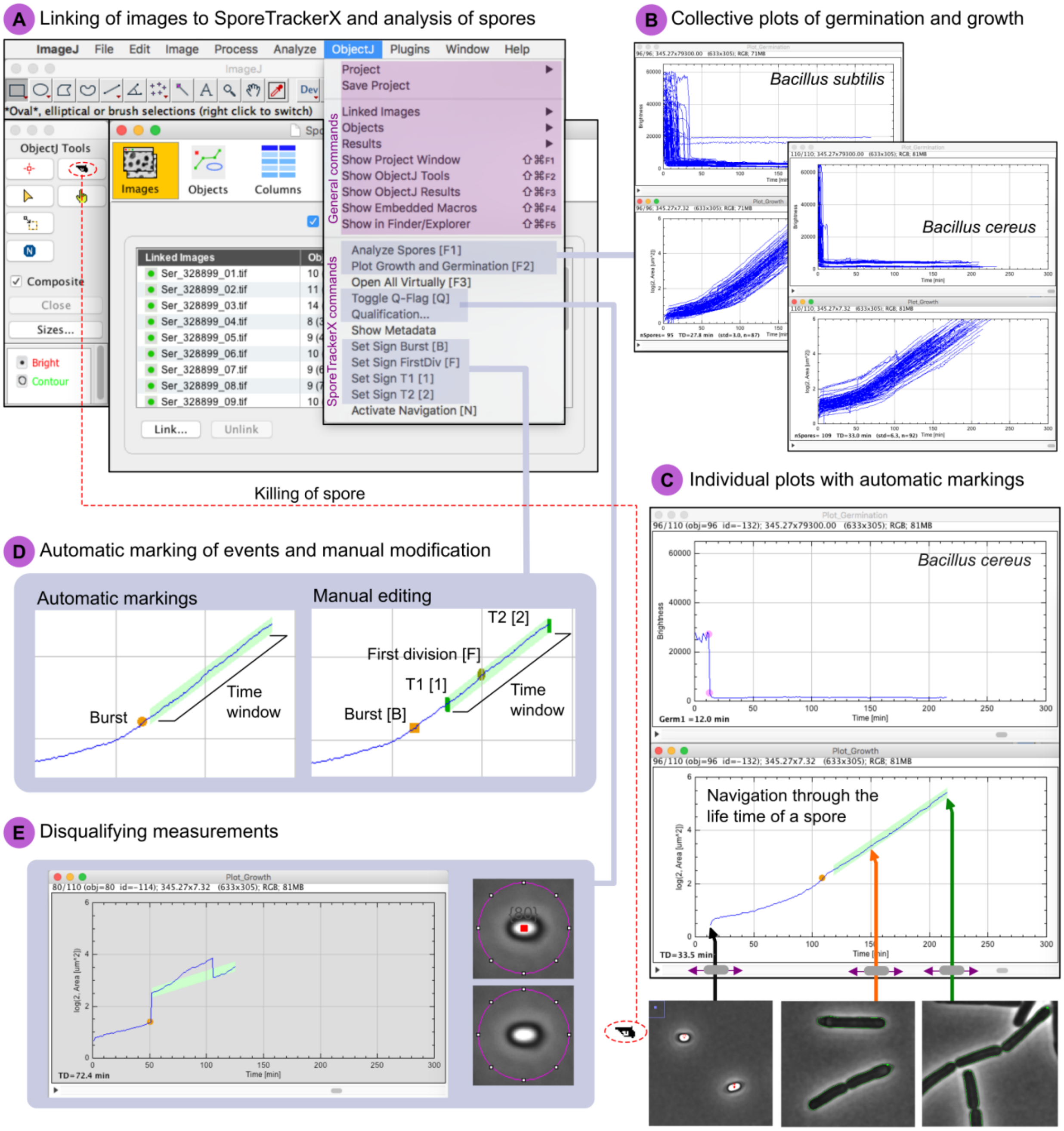

Figure 2. Overview of the data analysis of spores using SporeTrackerX. A) When opening the SporeTrackerX.ojj file through ImageJ, the movies to be analysed can be dragged into the panel for "Linked Images". The ObjectJ menu shows general commands in the upper part, and below these the macro commands specific to SporetrackerX, starting with "Analyze Spores", which analyses all linked movies. B) During the analysis, a pair of plot windows for germination and growth are periodically updated.The plot windows show the superimposition of all plot lines

available so far. After Analysis, these plot windows can be re-created in absence of the image files. A plot window holds a stack of all plots plus one additional stack frame showing the superimposition of all. Here, plot pairs of two different experiments are shown. C) A click-and-drag operation in one of the plot windows will trigger real-time playback of the corresponding spore, following the cursor position along the time axis. Here, screenshots at three different cell ages are shown. D) If necessary, automatic measurements can be adjusted or corrected by locating the cursor on the plot's time axis and using one of the "Set Sign" command shortcuts. E) A situation is shown where the growth was not interpreted correctly. This spore can either be disqualified or removed completely from the entire project with the pistol tool.

6. Feed-back during analysis

Processing of many large timelapse movies requires time, and it is important that the user can verify correct operation without having to wait until the process is completed. Therefore, SporeTrackerX provides two different ways for feedback. First, a collective plot is permanently displayed on the screen and updated after each of the ~20 movies per project file has been processed, showing a superimposition of all growth curves that have been found so far. Secondly, a controlled interruption option is available that can be invoked by keeping the Caps Lock key down. Image processing is then halted in a safe state, and the user can inspect all images, plots and results, and subsequently choose to continue safely or otherwise to abort the process. However, no windows should be closed during inspection.

7. Handling of plots and advanced navigation

SporeTrackerX.ojj project files store the image links, macros, markers and results, which enables the user to re-build plots or derive results at a later time, without opening the linked images. The plot windows "Germination vs time" and "Growth vs time" can be shown vertically tiled on the screen with their common time axis being aligned. Each of these windows accomodates a stack of as many plots as there are detected spores, plus an additional frame at the end of the stack containing a collective plot as superimposition of all plots (Fig. 2 B). Using the special

navigation tool in ObjectJ's tool window, the user can activate either of these plot windows and navigate back and forth along the time axis, while the growth of the corresponding spore or colony is being played back accordingly in the linked virtual movie (Fig. 2 C). In the collective plot, one of the many curves can be selected to isolate it for further inspection, which is typically done to study outliers.

While SporeTrackerX creates plots, it detects certain events automatically and derives the desired numerical results. In an interactive step, automatic results can be cleared or changed via several "Set Sign" shortcuts, that toggle the correponding sign on or off at the current cursor position. Signs can be set or reset for "First Division", "Burst", and for the time window from "T1" to "T2", which is used to calculate the generation time (TD) (Fig. 2 D). There is also the option to disqualify an undesired object from the data set (Fig. 2 E). Disqualified objects do not contribute to statistical evaluation and optionally can be deleted completely.

8. Results table

In the ObjectJ result table, each row is dynamically connected to an object and contains data about the life history of the corresponding spore and its outgrowing microcolony (Fig. 3). Double-clicking a row immediately shows the corresponding phase-bright spore in frame #1, from where further growth can be followed in time. Generally, any number of columns, either for strings or for numerical data, can be appended to the linked results table. In SporeTrackerX, such columns are created and populated during the analysis of the spores, and are updated after manual interaction. Appearance, color and visibility of a column can be adjusted for visual clarification. Basic statistics can optionally be shown in additional rows below the column title, and histograms can be readily created and modified. For the overall verification of the acquired data, a column can be sorted to focus on extreme values typically caused by outliers or artifacts. ObjectJ's qualification feature allows to define population subsets by applying criteria that can be edited in the Qualifiers panel or on macro level. Only qualified objects contribute to statistics, histograms and sorting. Any set of results can be exported as delimited text for post-processing in other applications. Plots, histograms or other data can be re-created from the newest project data at any time and are typically not saved as individual files, which keeps the file structure

simple and the desktop clean. Only the project file next to the linked movies appears in an experiment's folder, which makes the inter-connection of the files self-explanatory without having to open an application.

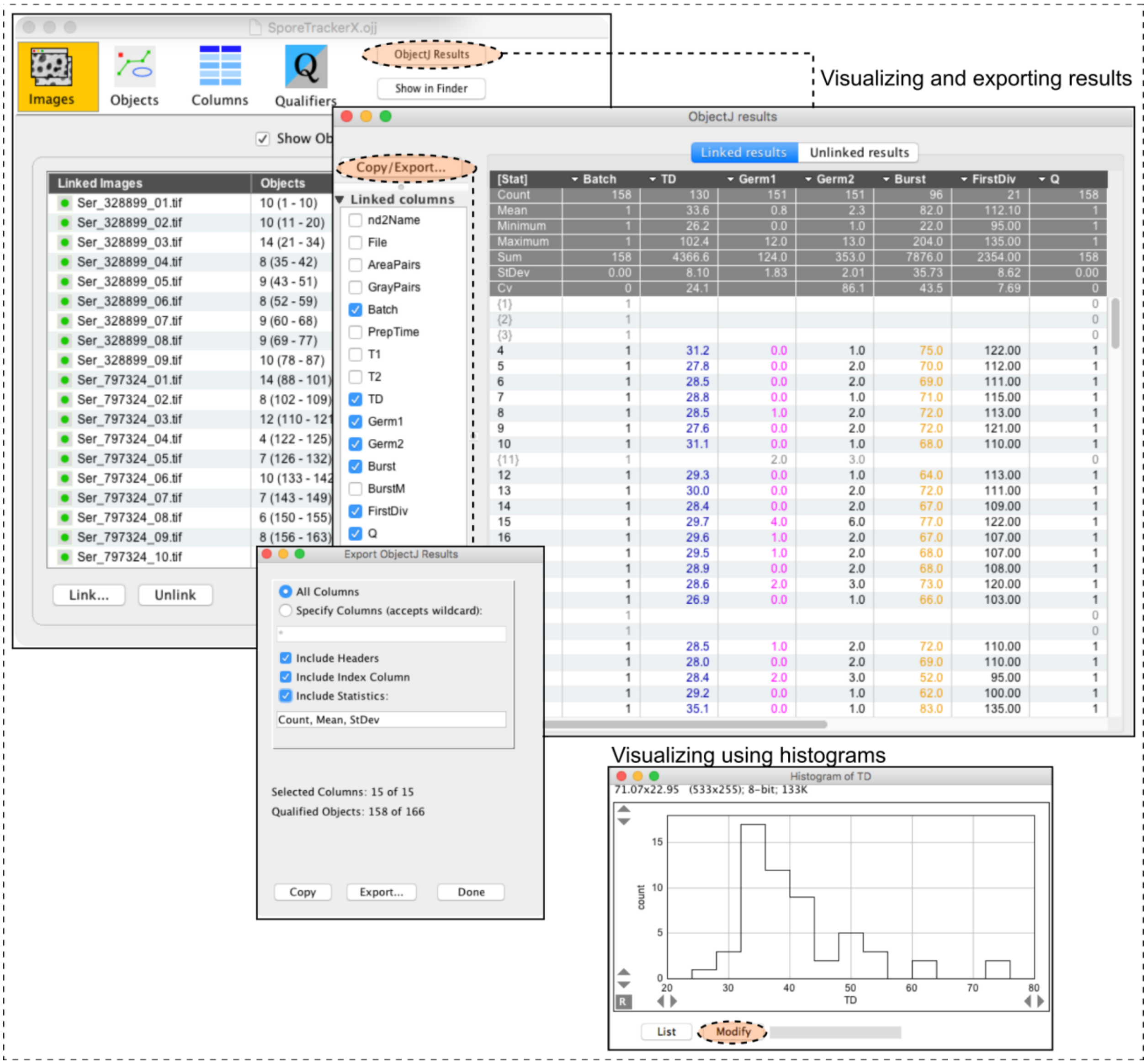

Figure 3. Retrieving results. After the data analysis the results can be viewed. The panel "Linked columns" lists all available column titles and controls their visibility. Data of unqualified objects appear gray and do not contribute to statistics. All result columns, or a subset

which can be defined by wildcards, can be exported as delimited text to other applications. Via contextual menus, histograms can be generated and modified if desidered.

9. Back-up strategy

Before generating data in the range of hundreds of gigabytes, a backup strategy has been considered in advance. For the entire set of experiments, we used two external 5TB USB3 disks, one for analysis and one for safety backup without further activity. This way, the acquired movies existed twice, and further analysis could take place on any computer without occupying its internal disk. During the analysis, the individual project files accumulate information that is gained automatically or interactively, while the movies remain unchanged. Consequently, only the up-to-date versions of the project files need to be backed-up. For this, a utility macro was written to create a "Shadow Directory [https://sils.fnwi.uva.nl/bcb/objectj/examples/utils/ShadowDirectory], which duplicates the entire directory tree including all ".ojj" project files, but without copying the images or other file types. It takes into account that the meaning of a file may only be clear if its position in the tree is known. Such a shadow directory can be easily moved e.g. to a USB stick or to a cloud service, as it is relatively small. Still the directory structure is intact, and it can be re-united any time with a directory tree holding the original images. Besides simplified back-up, the shadow directory gives the user the freedom to perform many tasks "off-line" in absence of the large data set that cannot be copied. These tasks include the generation of growth and germination plots, qualifying subsets, deleting objects, observing, creating and exporting histograms or derived result columns, and saving the updated ".ojj" project file which will show the correct overlay when later being re-united with the images.

10. Hardware and storage space

Image acquisition was performed on a Nikon Eclipse Ti microscope equipped with the NIS elements software version 4.50.00. The resulting very large ".nd2" files were then converted to ".tif" movies and stored in the corresponding directory trees on the two external drives, after

which the occupied storage space on the microscope could be released. Further analysis took place under MacOS El Capitan 10.11.3 (with Intel Core i5 CPU and 8 GB RAM). The program has been tested on MacOS and Windows OS. Table 1 shows figures that correspond approximately to the experiments described above. Analysis of one movie with ~10 spores lasted ~1 minute.

Table 1 An example of the storage space for a set of single-channel experiments

**Storage**	
Number of image folders (experiments)	12
Movies per folder	20
Frames per movie	400
MB per frame and channel (16-bit)	8
Total storage for entire set of experiments (1 channel)	750 GB

11. Conclusion

When processing complex data sets of large timelapse movies, aspects of file organisation, storage on external media, back-up methods, processing virtual stacks and interactive navigation need to be carefully optimized. With SporeTrackerX we present a method that analyses germination and outgrowth of bacterial spores and addresses these themes. It has been successfully tested with data close to the terabyte range of *Bacillus cereus* and *Bacillus subtilis* spores (in this article), but also of *Bacillus weihenstephanensis* spores (unpublished data). Rather than creating a new ImageJ plugin with the necessity to anticipate and include all needed features and to add the corresponding dialog boxes for user input, we chose a different strategy. By using the plugin ObjectJ, which provides an additional level of flexibility due to the concept of embedded macros, it was possible to realise the desired functionality as macro code. The required code is stored locally in a project file and can easily be displayed and modified. Many settings appear as a well-documented assignments of global variables that can be changed or communicated faster and with better overview than in dialog boxes. It also allows fine-tuning of user-specific requirements, shortcut keys and data output even by a user with minimal programming skills. Additionally, ObjectJ's dynamic link between images, overlays, results and plot commands provides integration of all components while the code remains compact and can

be easily exposed and edited. For example, future experiments may require more sophisticated thresholding methods or transient preprocessing, which is not implemented before the problems are known. Special care has been taken to allow the user to browse through the lifetime of any spore and focus on individual events without noticeable time delay even if the acquired movies are approaching the terabyte range. In conclusion, we present SporeTrackerX that will enable the rapid analysis of data acquired through live imaging, thus contributing to the understanding of the physiology and morphogenesis of spore-forming bacteria. Finally, the data generated contribute to the generation of predictive models for growth of pathogenic spore-formers that are of concern in the food industry and healthcare.

Acknowledgements

S. Omardien acknowledges the Erasmus Mundus Action 2 program (EMA2) and University of Amsterdam for funding. The authors declare that they have no conflict of interest.

References

1. Nicholson, W. L., Munakata, N., Horneck, G., Melosh, H. J. & Setlow, P. Resistance of Bacillus endospores to extreme terrestrial and extraterrestrial environments. *Microbiol. Mol. Biol. Rev.* **64,** 548–72 (2000).

2. Setlow, P. Spores of Bacillus subtilis: Their resistance to and killing by radiation, heat and chemicals. *J. Appl. Microbiol.* **101,** 514–525 (2006).

3. Setlow, P. Spore resistance properties. *Microbiol Spectr.* **2,** 1–14 (2014).

4. Wells-Bennik, M. H. J. *et al.* Bacterial spores in food: Survival, emergence, and outgrowth. *Annu. Rev. Food Sci. Technol.* **7,** 457–482 (2016).

5. CDC. *Antibiotic resistance threats in the United States. U.S Department of Health and Human* (2013). doi:CS239559-B

6. Binkley, C. E., Cinti, S., Simeone, D. M. & Colletti, L. M. Bacillus anthracis as an agent of bioterrorism: A review emphasizing surgical treatment. *Ann. Surg.* **236,** 9–16 (2002).

7. Rosales-Mendoza, S. & Angulo, C. Bacillus subtilis comes of age as a vaccine production host and delivery vehicle. *Expert Rev. Vaccines* **14,** 1–14 (2015).

8. Potot, S., Serra, C. R., Henriques, A. O. & Schyns, G. Display of recombinant proteins on Bacillus subtilis spores, using a coat-associated enzyme as the carrier. *Appl. Environ. Microbiol.* **76,** 5926–5933 (2010).

9. Bader, J., Albin, A. & Stahl, U. Spore-forming bacteria and their utilisation as probiotics. *Benef. Microbes* **3,** 67–75 (2012).

10. Setlow, P., Wang, S. & Li, Y.-Q. Germination of spores of the orders Bacillales and Clostridiales GCW: Spore germ cell wall. *Annu. Rev. Microbiol.* **71,** 459–477 (2017).

11. Pandey, R. *et al.* Live cell imaging of germination and outgrowth of individual bacillus subtilis spores; the effect of heat stress quantitatively analyzed with SporeTracker. *PLoS One* **8,** e58972 (2013).

12. Pandey, R. *et al.* Quantitative analysis of the effect of specific tea compounds on germination and outgrowth of Bacillus subtilis spores at single cell resolution. *Food Microbiol.* **45 Pt A,** 1–8 (2014).

13. Vischer, N. O. E. *et al.* Cell age dependent concentration of Escherichia coli divisome proteins analyzed with ImageJ and ObjectJ. *Front. Microbiol.* **6,** 1–18 (2015).

14. Syvertsson, S., Vischer, N. O. E., Gao, Y. & Hamoen, L. W. When phase contrast fails: ChainTracer and NucTracer, two ImageJ methods for semi-automated single cell analysis using membrane or DNA staining. *PLoS One* **11,** 1–11 (2016).
